# Contribution of analog signaling to neurotransmitter interactions and behavior: Role of transporter‐mediated nonquantal dopamine release

**DOI:** 10.14814/phy2.15088

**Published:** 2021-11-11

**Authors:** Viktor Román, Rita Kedves, Kristóf Kelemen, Zsolt Némethy, Beáta Sperlágh, Balázs Lendvai, E. Sylvester Vizi

**Affiliations:** ^1^ Pharmacology and Drug Safety Research Gedeon Richter Plc. Budapest Hungary; ^2^ Institute of Experimental Medicine Budapest Hungary; ^3^ Department of Pharmacology and Pharmacotherapy Semmelweis University Budapest Hungary

**Keywords:** cytoplasmic release, dopamine, nonquantal, social behavior, transporter

## Abstract

Neuronal networks cause changes in behaviorally important information processing through the vesicular release of neurotransmitters governed by the rate and timing of action potentials (APs). Herein, we provide evidence that dopamine (DA), nonquantally released from the cytoplasm, may exert similar effects in vivo. In mouse slice preparations, (+/−)‐3,4‐methylenedioxy‐methamphetamine (MDMA, or ecstasy) and β‐phenylethylamine (β‐PEA)‐induced DA release in the striatum and nucleus accumbens (NAc), two regions of the brain involved in reward‐driven and social behavior and inhibited the axonal stimulation‐induced release of tritiated acetylcholine ([^3^H]ACh) in the striatum. The DA transporter (DAT) inhibitor (GBR‐12909) prevented MDMA and β‐PEA from causing DA release. GBR‐12909 could also restore some of the stimulated acetylcholine release reduced by MDMA or β‐PEA in the striatum confirming the fundamental role of DAT. In addition, hypothermia could prevent the β‐PEA‐induced release in the striatum and in the NAc. Sulpiride, a D2 receptor antagonist, also prevented the inhibitory effects of MDMA or β‐PEA on stimulated ACh release, suggesting they act indirectly via binding of DA. Reflecting the neurochemical interactions in brain slices at higher system level, MDMA altered the social behavior of rats by preferentially enhancing passive social behavior. Similar to the in vitro effects, GBR‐12909 treatment reversed specific elements of the MDMA‐induced changes in behavior, such as passive social behavior, while left others including social play unchanged. The changes in behavior by the high level of extracellular DA–– a significant amount originating from cytoplasmic release––suggest that in addition to digital computation through synapses, the brain also uses analog communication, such as DA signaling, to mediate some elements of complex behaviors, but in a much longer time scale.

## Graphical abstract

We show that DA released by MDMA or β‐PEA via the reverse operation of the DA transporter (DAT) ––a mechanism not coupled to axonal firing––is able to affect local cholinergic transmission in the striatum by a nonsynaptic mechanism. DA of cytoplasmic origin, released by DAT reversal, influences distinct elements of social behavior induced by MDMA as shown by the in vivo effects of selective DAT inhibition on passive social behavior induced by MDMA, while other components of the MDMA‐induced social behavior, such as reduced social play was not changed, highlighting DA‐mediated‐specific social effects. Our findings demonstrate an example for the in vivo role of nonquantal DA release through transporter reversal.

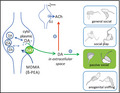

## 1 INTRODUCTION

The vertebrate central nervous system must simultaneously perform numerous computations via axonal firing. The arrival of action potentials (APs) at nerve terminals results in Ca^2+^ influx and leads to activity‐dependent exocytotic neurotransmitter release into the synaptic cleft. The key behavioral role of synapses activated by series of presynaptic APs in chemical transmission has been generally accepted. Furthermore, neurochemical evidence has shown that monoamine transporters are able to function in reverse and release transmitters from the cytoplasm (Sulzer et al., 1995) independently of axonal firing and external Ca^2+^ (Raiteri & Raiteri, 2015). Substrates of dopamine (DA) transporters (DATs), such as amphetamines and β‐phenylethylamine (β‐PEA), can regulate transporter trafficking, reduce transporter capacity, and promote carrier‐mediated DA efflux from the cytoplasm (Chi & Reith, 2003; Gudelsky & Yamamoto, 2008; Saunders et al., 2000; Sitte et al., 1998; Sulzer et al., 1993, 1995; Wheeler et al., 2015; Zsilla et al., 2018). Important progress has been made due to the recent observation that revealed a correlation between the effects of amphetamine on DAT trafficking in vitro and hypermotility in vivo (Wheeler et al., 2015); in particular, the activation of D1/D5 receptors coupled to protein kinase A inhibits both DAT internalization and behavior in response to amphetamine administration. Under these conditions, the DAT functions in reverse and plays a critical role in releasing DA from the cytoplasm. Indeed, DAT‐knockout mice are insensitive to amphetamine to release DA from striatal slices (Jones et al., 1998) and to produce increase in motility (Giros et al., 1996).

It is also recognized that quantal release of endogenous DA in the striatum could prevent acetylcholine (ACh) release following electrical stimulation of the substantia nigra (Bartholini et al., 1976); however, in slice preparations, a significant increase in ACh release was detected if the nigrostriatal pathway had been previously impaired suggesting a tonic inhibition by DA (Lendvai et al., 1993; Vizi et al., 1977). These findings indicate that the DA released in response to axonal firing can result in functional interactions between dopaminergic and cholinergic neurons in the striatum and affect release through the activation of D2 heteroreceptors (Brooks et al., 2007). However, no observations have been made regarding the mechanism by which transmitters released from the cytoplasm exert similar effects on brain areas where no synaptic contacts are found between dopaminergic and cholinergic neurons. We focused our neurochemical studies on two important sites in the brain, namely, the striatum and the nucleus accumbens (NAc), that play central roles in reward‐driven and social behavior. The striatum and the NAc are densely innervated by dopaminergic axons with boutons that form a few symmetric synapses onto medium spiny output neurons (Smith & Bolam, 1990; Descarries et al., 1996) but virtually no synaptic contacts with other axonal varicosities (Sesack & Pickel, 1990; Descarries et al., 1996; Moss & Bolam, 2008). It is known that (+/−)‐3,4‐methylenedioxy‐methamphetamine (MDMA, or ecstasy), similar to the endogenous trace amine β‐phenylethylamine (β‐PEA), releases DA from the cytoplasm by the reverse function of DATs (Janssen et al., 1999; Zsilla et al., 2018) via a mechanism that is independent of neuronal activity and tetrodotoxin (TTX) application (Zsilla et al., 2018). Uptake inhibition also contributes to the effect of MDMA on DA outflow (Iravani et al., 2000). Therefore, one aim of our study was to examine the effects of the MDMA‐ and β‐PEA‐induced nonquantal release of cytoplasmic [^3^H]DA on the stimulation‐induced release of [^3^H]ACh in ex vivo slice preparations. Both compounds‐induced substantial DA release from nigrostriatal and accumbal terminals and inhibited ACh release in the striatum. Inhibition of DAT activity by the nonsubstrate inhibitor GBR‐12909 prevented MDMA from inducing DA release in the striatum and NAc and from ß‐PEA from inducing DA release in the NAc. Moreover, hypothermic conditions known to inhibit transporter activity (Bonnet et al., 1990) attenuated ß‐PEA from inducing DA release in both regions as well as MDMA‐ induced release in the NAc. Importantly, preventing cytoplasmic DA release by DAT inhibition attenuated the impact of D2R‐dependent inhibition of stimulated ACh release. At higher system level, it is generally accepted that social behaviors (Vanderschuren et al., 2016) are regulated by the mesolimbic dopaminergic pathway through the release of DA in innervated areas, such as the striatum and NAc (Alcaro et al., 2007; Arias‐Carrión et al., 2010), and MDMA has prosocial effects in nonhuman primates (Pitts et al., 2017) and facilitates passive social behavior in rats (Ando et al., 2006). Therefore, driven by the neurochemical effects of the selective DAT inhibitor understood in a slice preparation, our aim was to explore the in vivo role of the MDMA‐induced nonquantal DA release through studying the social effects of this drug.

## 2 METHODS

### 2.1 Release of neurotransmitters

The release of [^3^H]DA or [^3^H]acetylcholine ([^3^H]ACh) was measured using ex vivo acute slice preparations from the striatum and NAc. Male CD1 mice weighing 20–28 g that were bred in the local animal facility were used in this study. Animals received food and water ad libitum, and lighting was maintained on a 12 h cycle. The experiments were conducted in the dark cycle in strict accordance with institutional guidelines (Institute of Experimental Medicine), including the European Directive (2010/63/EU). The mice were anesthetized with ether and decapitated, and the brain was quickly removed and placed into ice‐cold Krebs solution. Unless otherwise indicated, all the experiments were performed at 37°C in modified Krebs solution (pH 7.4) containing 118 mM NaCl, 4.7 mM KCI, 2.5 mM CaCl_2_, 1.2 mM KH_2_PO_4_, 1.2 mM MgSO_4_, 25 mM NaHCO_3_, and 12.5 mM glucose that was continuously saturated with 95% O_2_ + 5% CO_2_. Then, the striatum or NAc was harvested and sliced into 400‐μm‐thick sections (we gratefully acknowledge the help of Dr. M. Palkovits). The release experiments were performed as previously described (Milusheva et al., 2008; Zsilla et al., 2018). Briefly, the slices were incubated in 1 ml of Krebs solution containing 5 μCi/ml [^3^H]DA or methyl‐ [^3^H]choline for 45 min. After incubation, the slices were transferred to tissue chambers and continuously perfused with modified Krebs solution at a rate of 0.5 ml/min. After 60 min of preperfusion, samples were collected every 3 min and assayed for [^3^H]DA or [^3^H]ACh. For the optimal detection of [^3^H]DA release, to prevent the metabolism of catecholamines by monoamine oxidase (MAO) during the release experiments, the Krebs solution contained 10 µM pargyline. For the detection of [^3^H]ACh release, striatal slices were incubated for 60 min in a 2‐ml organ bath at 37°C in Krebs solution. To selectively prevent choline reuptake by the high‐affinity choline transporter inhibitor, hemicholinium‐3 (10 µM) was present throughout the experiments; it had no effect on the resting and axonal stimulation‐induced release of ACh. The total radioactivity released from the tissue and collected in the superfusate was considered to represent the amount of [^3^H]ACh released and not that of [^3^H]choline (Wikberg, 1977). At the end of radioactivity collection, the residual radioactivity in the preparations was extracted with 1 ml TCA (10%) for 120 min and measured using an aliquot (0.1 ml) of the supernatant. The radioactivity present in the tissues and collection samples were measured by a Packard‐Canberra TR 1900 liquid scintillation counter, and the [^3^H]ACh or [^3^H]DA content was normalized to the Bq/g tissue.

The release of radioactivity was elicited during two periods (S1 and S2) using field stimulation (120 pulses at 2 Hz, impulse duration of 2 ms, using a Grass S88 stimulator from Astro‐Med), that occurred during the 3rd and 18th collections. Using a custom‐made equation, the radioactivity measured in the samples was calculated as the fractional release (FR% = released tritium in Bq/g × 100/tritium in Bq/g tissue at the time the samples were measured). The effect of axonal stimulation or drugs on the release was evaluated as the ratio of the area under the curve of the total radioactivity release to the resting release in response to the first and second stimulation (FRS2/FRS1). The release induced by the first stimulation was considered the internal standard. A similar calculation was performed for the resting release (FRR2/FRR1). Unless otherwise stated, the drugs were administered between the two electrical stimulations, and the ratio of the FR values due to R1 (average resting release from the first and second samples) and R2 (average resting release from the 10th and 11th fractions) was used to demonstrate the effects on resting release. The release of both neurotransmitters induced by field stimulation was 1 µM TTX‐sensitive and [Ca^2+^]_o_‐dependent. By contrast, the resting and β‐PEA‐ or MDMA‐induced increases in DA release was [Ca^2+^]_o_‐independent (Zsilla et al., 2018). 6–9 distinct slices were examined per treatment group.

**FIGURE 1:**
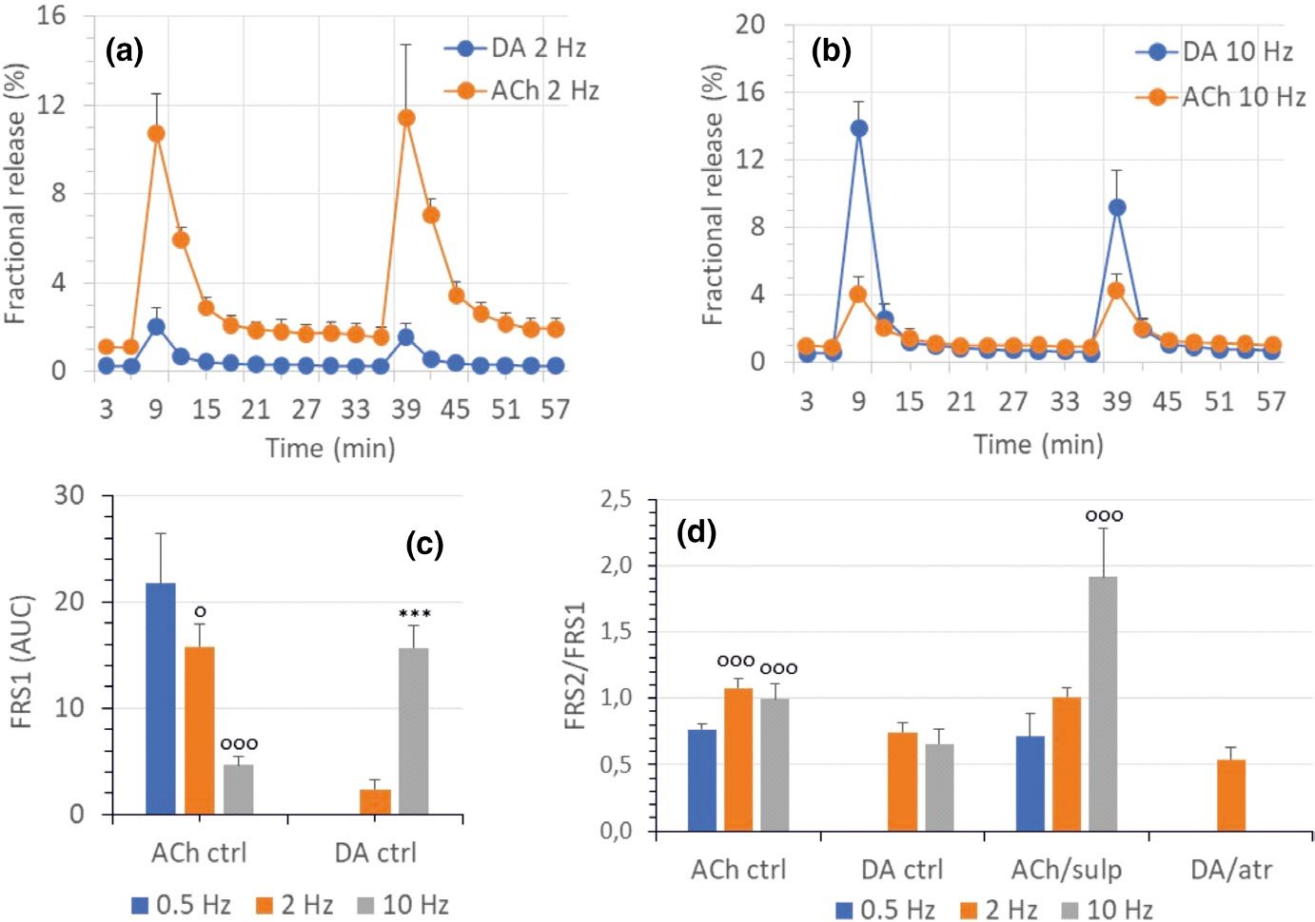
Release of ACh and DA in the striatum in response to various frequencies of electrical stimulation. The firing frequency of dopaminergic neurons varies from low (<5) to brief bursts of high frequency (>10 Hz) stimulation. The same number of shocks (120) were applied. (a) Stimulation at 2 Hz induces increased release of ACh, while (b) at 10 Hz frequency, it supports DA release. (c) Area under the curve analysis shows the frequency‐dependent decrease in ACh release, while the opposite is seen for DA. (d) Modulation of frequency‐dependent release. Sulpiride at 10 µM applied from the 24th min increases ACh release only at high frequency and fails to influence ACh release at 2 Hz. Note that using the same number of shocks (120) at 2 Hz, DA was released in 60 s while at 10 Hz only 12 s was needed. In the latter case, considering that the concentrations of transmitters, drop during diffusion, the ambient concentration of DA could have been much higher. At a 2 Hz stimulation frequency the concomitantly released DA does not reach as high a concentration as it does at 10 Hz and therefore does not affect the release of ACh. Atropine at 10 µM had no effect on DA release. Data represent the mean + SD. ^o^0.5 Hz vs. 2 or 10 Hz (ANOVA, ^o^ *p* < 0.05, ^ooo^ *p* < 0.001); or *2 vs. 10 Hz (*t*‐test, ***p < 0.001)

In a separate set of experiments, 0.5, 2, and 10 Hz of electrical stimuli were used to determine the optimal stimulation frequency for DA and ACh release. Low‐frequency electrical field stimulation (2 Hz) of the striatal slices resulted in low release of DA, while ACh release was predominant. In contrast, at a high frequency (10 Hz), the opposite was observed; the electrical stimulation‐induced DA release increased, as shown by the area under the curve values for S1 (Figure 1a–c). DA D2 receptors, which block ACh release in the striatum under normal circumstances, were antagonized by sulpiride (10 µM). Sulpiride increased the stimulation‐induced release of ACh only at a frequency of 10 Hz (Figure 1d) and not at 2 Hz frequency, indicating that D2 receptors mediate the inhibitory effect of DA release on ACh release. However, this enhanced quantal release of ACh was seen only when high frequency (10 Hz) stimulation was used, showing that the diffusion time and biophase DA concentration around the cholinergic axon terminals are important. In our previous experiments (Milusheva et al., 2008; Zsilla et al., 2018), high‐pressure liquid chromatography was used to validate the tritiated DA release. The data showed that the release of radioactivity from brain slices loaded with [^3^H]DA was mostly due to DA (>85%) and its metabolites of cytoplasmic origin (DOPAC, DOPET, and DOPAL <15%).

Transporter proteins, including DAT, are sensitive to temperature (Zsilla et al., 2018). At low temperature, only action potential‐induced quantal release occurs, and carrier‐mediated resting release is inhibited (Sotnikova et al., 2004; Vizi, 1998). The temperature of the Krebs solution was quickly changed with a thermoelectric device between 37 and 17°C (Frigomix, B Braun) and kept constant.

### 2.2 In vivo social behavior

Three cohorts of male Wistar rats (Toxicoop Ltd.) were used (*n* = 144 rats in total) for the in vivo experiments. The rats arrived at the local vivarium on postnatal day 21 (shortly after separation from their mothers) and were subjected to behavioral observations 2 weeks later; the rats weighed 75–150 g. The animals were housed in polycarbonate cages in groups of four in a thermostatically controlled room at 21 ± 1°C. The room was artificially illuminated from 6 a.m. to 6 p.m. The rats were fed conventional laboratory rat food (sniff R/M + H Spezieldiäten GmbH D‐59494 Soest). The three cohorts of rats were used for three separate measurements; the dose‐dependent effect of MDMA was studied in the first cohort, the dose‐dependent effect of GBR‐12909 was studied in the second cohort, and finally, in the third cohort, the rats were subjected to a coadministration experiment using selected doses of MDMA, GBR‐12909, or their combination. All of the procedures conformed to the guidelines of the National Institutes of Health for the care and use of laboratory animals and were approved by the Local Ethical Committee of Gedeon Richter Plc.; these experiments were also carried out in strict compliance with the European Directive 2010/63/EU regarding the care and use of laboratory animals for experimental procedures. All efforts were made to minimize the number of animals required and their suffering.

#### 2.2.1 Reciprocal dyadic social interaction test

The social interaction tests were performed in a rat cage (dimensions 37 × 37 × 19 cm) with a 2‐cm layer of sawdust on the bottom under dim light conditions (approximately 12 lux). One day prior to the test, the rats were acclimated to human handling and to the testing environment by individually placing them in the arena for 5 min. After acclimation, the rats were isolated to induce intense social behavior during testing the next day. On the test day, weight‐matched pairs of rats that were unfamiliar with each other were simultaneously placed in the opposite corners of the testing cage. Videos were captured to record the behavior of the rats (Ethovision XT13, Noldus) for 15 min, and these videos were subsequently analyzed by a blinded human observer. The analysis of behavior included the detailed classification of social behavior into the categories of passive social behavior, social play behavior, anogenital sniffing, and general social behavior. Passive social behavior included lying adjacent and ambulating in close contact. Social play behavior consisted of pinning (imitation, playful submissive‐dominant behavior) and pouncing (nape attack, mounting). Anogenital sniffing was defined as the intensive investigation of another rat's genitals and anal region, which is a means of identification among rats. General social behavior included all other forms of social behavior, such as sniffing of the facial area or allogrooming. The duration of all instances of the four components of social behavior (namely, passive social behavior, social play, anogenital sniffing, and general social investigation) was summed to calculate the total social investigation time. One data point represents one pair of animals.

### 2.3 Drugs

The drug MDMA was dissolved in physiological saline (in a volume of 2 ml/kg) and administered at doses of 0.5, 2.5, and 5 mg/kg. Morphine (ICN Hungary Plc.) was also dissolved in physiological saline (in a volume of 2 ml/kg) and administered at a dose of 1 mg/kg 30 min before the social interaction assay. GBR‐12909 (Sigma) was dissolved in physiological saline (in a volume of 5 ml/kg) and administered at doses of 1, 3, and 10 mg/kg. All the drugs were administered by subcutaneous (s.c.) injection in the flank region 30 min prior to the behavioral observations. In the coadministration experiment, MDMA and GBR‐12909 were administered to the rats in the two flank regions within a short period of time 30 min prior to the observations. DA‐3,4‐[7,8‐3H(N)], with a specific activity of 60 Ci/mmol, and choline chloride [methyl‐3H], with a specific activity of 80 Ci/mmol, were purchased from American Radiolabeled Chemicals (ARC). β‐PEA was obtained from Sigma. All the other chemicals were obtained from Sigma.

### 2.4 Statistics

The data from the neurotransmitter release experiments were analyzed by Student's *t*‐test or one‐way ANOVA followed by Tukey's multiple comparisons test (GraphPad Prism 8). The level of significance was set to *p *= 0.05. Data are presented and means and SDs.

The social behavioral data were analyzed by *t*‐test or one‐way analysis of variance (ANOVA) followed by Tukey's or Dunnett's multiple comparisons test (GraphPad Prism 8). The level of significance was set to *p *= 0.05. Principal component analysis was carried out on the combined dataset. This method is a dimension reduction technique used to identify structure in the data without taking into account the treatment grouping (Multivariate Analysis in the Pharmaceutical Industry, 2018). PERMANOVA (permutational MANOVA) was used for partitioning sums of squares in order to compare centroids and spreads of treatment groups in the multivariate feature space (McArdle & Anderson, 2001). The method is widely used in ecology and genetics, where relatively few samples can be measured and response variables can be several thousands. New axes were constructed as linear combinations of the dependent variables that are orthogonal and explain the decrease in the total variance. Vehicle groups were merged after inspection of their homogeneity, and the doses of MDMA and GBR‐12909 were restricted to those used in the coadministration experiment. The data were scaled and centered, and a biplot was produced that showed the correlation of the behavior types with the new axes. Analysis was carried out using R statistical software (R Core Team, 2013; Venables & Ripley, 2002).

## 3 RESULTS

### 3.1 Neurochemical effects of nonquantal DA

**FIGURE 2:**
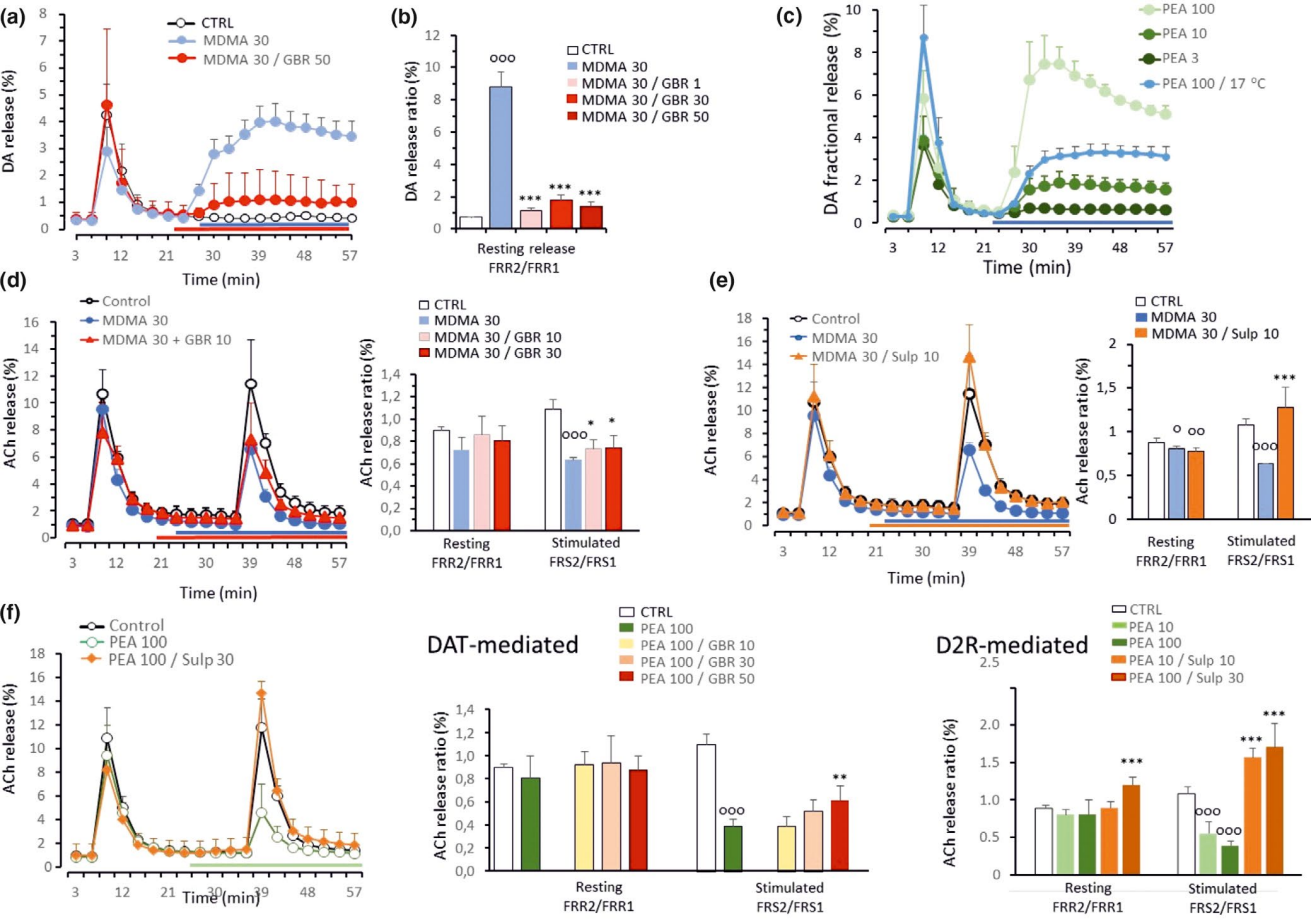
Effects of the cytoplasmic release of DA in response to MDMA and β‐PEA on neurotransmitter interactions in the striatum. Stimulation rate of 2 Hz (120 shocks) was applied. (a) In the absence of electrical stimulation, MDMA, when applied after the 24th min of perfusion, induces substantial release of DA under resting conditions that can be attenuated by GBR‐12909. (b) The MDMA‐induced DA release was blocked by 1, 30, and 50 µM GBR‐12909 under resting conditions. Note the extreme Y‐axis scale, which is due to the substantial resting release induced by MDMA. (c), The trace amine β‐PEA induced the release of DA in the striatum in a concentration‐dependent manner. Hypothermia inhibited the effect of β‐PEA at 100 µM, indicating the cytosolic origin of the released DA. (d), Electrical stimulation‐induced release of ACh in the striatum was indirectly attenuated by MDMA. The effect of GBR‐12909 (10 and 30 µM) was relatively small but reached significance; time‐lapse changes in the release (left) and release ratio values (right). (e) Sulpiride (10 µM) reversed the effect of MDMA (30 µM) on the striatal ACh release induced by electrical field stimulation, but it failed to interfere with MDMA under resting conditions. (f) Similar to MDMA, β‐PEA also inhibited the release of ACh in the striatum. The D2 antagonist sulpiride restored the inhibitory effect of PEA on the induced release of ACh. DAT‐mediated actions: the application of GBR‐12909 tended to prevent the inhibitory action of β‐PEA in a dose‐dependent manner but reached significance only at 50 µM. D2R‐mediated actions: sulpiride prevented the β‐PEA‐induced (10 and 100 µM) inhibition of ACh release at 10 and 30 µM. The resting release of ACh was not affected. The data are presented as the mean + standard deviation (SD). ^o^control versus MDMA or β‐PEA (^ooo^ *p* < 0.001; ^oo^ *p* < 0.01; ^o^ *p* < 0.05); * MDMA (30 µM) or β‐PEA (100 µM) versus GBR‐12909 (***p < 0.001, *p < 0.05); or sulpiride (***p < 0.001); (*n* = 6–9)

To start elucidating the potential neurotransmitter‐level mechanisms underlying the actions of MDMA, 30 µM MDMA was used to induce DA release from mouse striatal slices with 30 µM producing a much larger DA efflux that of 1 µM MDMA (Figure 2a). A selective DAT inhibitor, GBR‐12909, was used to demonstrate that the reverse function of the DAT was the underlying mechanism for the MDMA‐evoked release of DA. Indeed, 1, 30, or 50 µM GBR‐12909 mitigated the vast increase in DA release induced by MDMA under resting conditions (Figure 2a,b). In the striatum, the 30 µM MDMA‐evoked DA release at resting condition (in the absence of electrical stimulation) was very robust and maintained reaching a magnitude able to influence axonal stimulation‐evoked ACh release from a distant cholinergic neuron (see Figure 2e – sulpiride blocks MDMA‐evoked response).

Indeed, the MDMA‐induced changes in neurotransmitter release were not limited to DA in the striatum; the electrical field‐stimulated ACh response was also altered, and in particular, the ACh response at 2 Hz stimulation was significantly reduced by 30 µM MDMA (Figure 2d). When the release of [^3^H]ACh was measured at stimulation with 2 Hz in the absence of MDMA, the simultaneously released DA in the striatum exerted no inhibitory effects on ACh release while at 10 Hz stimulation frequency the ACh response was markedly decreased (Figure 1) suggesting that the inhibition by DA on the local ACh response gets stronger with the stimulation frequency. This is supported by the failure of sulpiride to affect ACh release at low frequencies (e.g., 2 Hz) (Figure 1d). Mirroring the neurochemical interactions to DA, at 2 Hz stimulation frequency, atropine failed to influence the release of DA (Figure 1d), indicating that there is no mutual interaction between dopaminergic and cholinergic terminals. Thus, when MDMA (30 µM) was also applied at 2 Hz stimulation, the reason for the change in the ACh response to the 2 Hz stimulation might be that the DA level after stimulation plus the additional cytoplasmic DA released by MDMA was high enough to inhibit the ACh release from local cholinergic terminals via D2 receptor activation (Figure 2e). Although, we did not apply a second electrical stimulus in the MDMA DA release experiments in this region, it is possible that when monitoring evoked ACh, concurrent DA release from vesicles during field stimulation could also influence axonal ACh release in the presence of MDMA. Given that dopamine terminals are connected to cholinergic targets in nonsynaptic ways this effect would also occur via extrasynaptic communication. Moreover, this evoked DA release is likely to play a smaller role compared to the large efflux of DA induced by DAT reversal (Figure 2a). The DAT inhibitor GBR‐12909 significantly attenuated the inhibitory effect of MDMA on ACh release confirming some role of cytoplasmic release in the inhibition of the ACh response (Figure 2d). A direct effect of MDMA on ACh release is unlikely because the release of ACh under resting conditions was not changed in accordance with the monoamine transporter‐specific mechanism of MDMA.

β‐PEA, which bears structural similarities to MDMA (phenethylamines), also inhibited the stimulation‐induced release of ACh according to the mechanism described above in a concentration‐dependent manner, further confirming an effect of cytoplasmic DA release on striatal ACh response (Figure 2f). GBR‐12909, at the highest concentration tested, partially restored the second field stimulation‐induced release of ACh that was inhibited by MDMA application (Figure 2f). The fact that sulpiride prevented β‐PEA from inhibiting the ACh release induced by axonal stimulation indicates an indirect effect of DA via D2 receptor activation (Figure 2f).

Similar to the effect of MDMA, the trace amine β‐PEA also triggered the release of DA from the cytoplasm under resting conditions in a concentration‐dependent manner (Figure 2c). The use of intermittent hypothermia, which blocks transporter activity (Bonnet et al., 1990; Liang & Rutledge, 1982), from the beginning of sample collection, effectively decreased the release of DA induced by 100 µM β‐PEA (Figure 2c). This finding also indicates the involvement of transporters in the effect of β‐PEA. In addition, the release induced by the first stimulation at low temperature was increased compared to the release induced by the first electrical stimulation, S_1_ at 37°C. These findings indicate that the transporter function was impaired thereby decreasing DA uptake and increasing extracellular levels of DA (Figure 2c).

**FIGURE 3:**
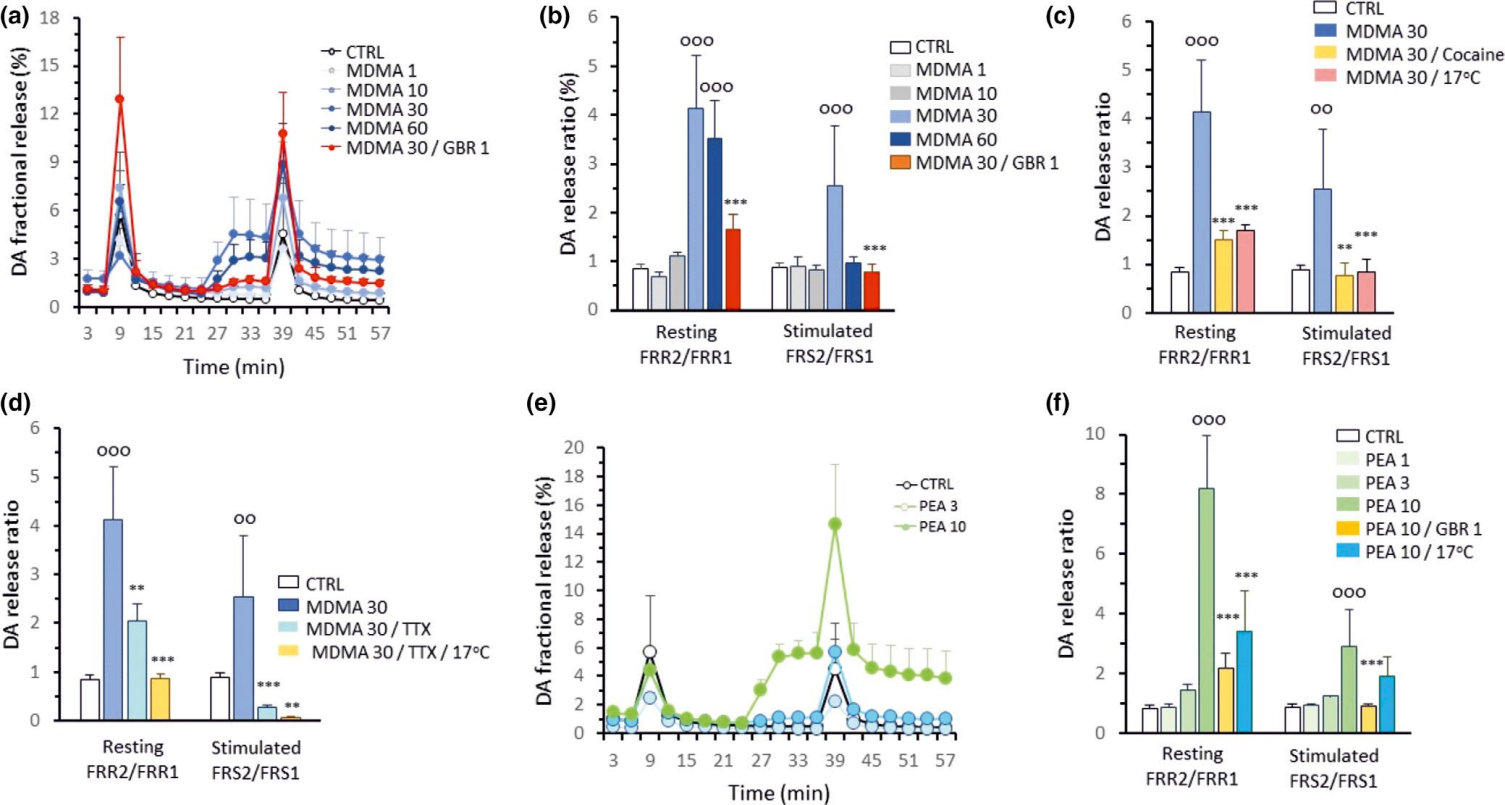
Modulation of nonquantal DA release in the NAc. Stimulation rate of 2 Hz (120 shocks) was applied. (a) Temporal dynamics of cytoplasmic DA release in response to different concentrations of MDMA (the inhibitory effect of GBR‐12909 is also shown). (b) Dose‐dependent release of DA in response to MDMA (maximal DA release by 30 µM MDMA was inhibited by 1 µM GBR‐12909). (c), The MDMA‐induced DA release was inhibited by cocaine (30 µM). Hypothermia was also inhibitory, indicating the cytoplasmic origin of the released DA. Cocaine and hypothermia also inhibited stimulated DA release in the presence of MDMA. (d) TTX (1 µM) inhibited the action of 30 µM MDMA, and this effect was further potentiated by hypothermia. (e) Temporal dynamics of the β‐PEA‐induced DA release in the NAc. (f) β‐PEA‐induced marked and concentration‐dependent release of DA from the NAc, and this effect was inhibited by the application of 1 µM GBR‐12909 throughout the experiment. Hypothermia inhibited the β‐PEA‐induced DA release only under resting conditions. The data are presented as the mean + SD. ^o^control versus MDMA or β‐PEA (^ooo^ *p* < 0.001; ^oo^ *p* < 0.01), *MDMA (30 µM) or β‐PEA (10 µM) versus inhibitors (***p < 0.001, **p < 0.01); (*n* = 6–9)

Using a NAc preparation, similar to the results obtained in the striatal slices, MDMA (1–60 µM)‐induced DA release under resting conditions in a concentration‐dependent manner with the maximal effect observed at a concentration of 30 µM (Figure 3a,b). In this region, we focused on the release of DA. Electrical field stimulation‐induced DA release in the NAc was also examined because the amount of DA released in this region was smaller than that in the striatum (see MDMA 30 µM). We found that the stimulation‐induced release of DA was potentiated by the application of 30 µM but not 60 µM MDMA. The resting / stimulated release of DA induced by 30 µM MDMA was inhibited by 1 µM GBR‐12909 (Figure 3a,b). It should be noted that a greater DA release was induced by the first electrical stimulation when GBR‐12909 was perfused from the beginning of the experiment, indicating that the inhibition of DAT increased the stimulation‐induced release in the absence of MDMA (Figure 3a) due to uptake inhibition. Hypothermia was also effective in reducing the MDMA‐induced release of resting and stimulated DA (Figure 3c), suggesting a role of transporter‐mediated actions. Cocaine, a nonsubstrate DAT inhibitor, also prevented the MDMA‐induced potentiation of DA release in the NAc slices, further supporting a role of transporters (Figure 3c). TTX alone could not fully block the MDMA‐induced DA release under either resting or stimulated conditions, while hypothermia further potentiated its inhibitory effect (Figure 3d) until a complete block of stimulation‐induced release was achieved.

As seen in the striatum, β‐PEA (10 μM) could also induce a large release of DA from the cytoplasm in the NAc under resting conditions while the effects of lower concentrations of 1 and 3 µM were not significant (Figure 3e). GBR‐12909 (1 µM) and hypothermia significantly reduced the release of DA induced by 10 µM β‐PEA under resting conditions while the enhanced release of DA was inhibited only by GBR‐12909 under stimulated conditions (Figure 3e,f).

### 3.2 Prevention of cytoplasmic DA release alters specific behavioral patterns induced by MDMA

Next, we investigated the impact of nonquantally released DA induced by the reverse function of transporters at a higher system level. It is known that treatment of adolescent rats with MDMA increases passive social behavior and decreases social play and general social behavior (Ando et al., 2006). We succeeded in proving (see Figures 2 and 3) that the cytoplasmic release of DA induced by MDMA could be prevented in vitro by DAT inhibition. Accordingly, we investigated in vivo the DAT inhibitor GBR‐12909 to alter MDMA‐induced social behavior. Morphine was used to verify that social behavior of rats is sensitive to a prosocial drug in the expected way.

**FIGURE 4:**
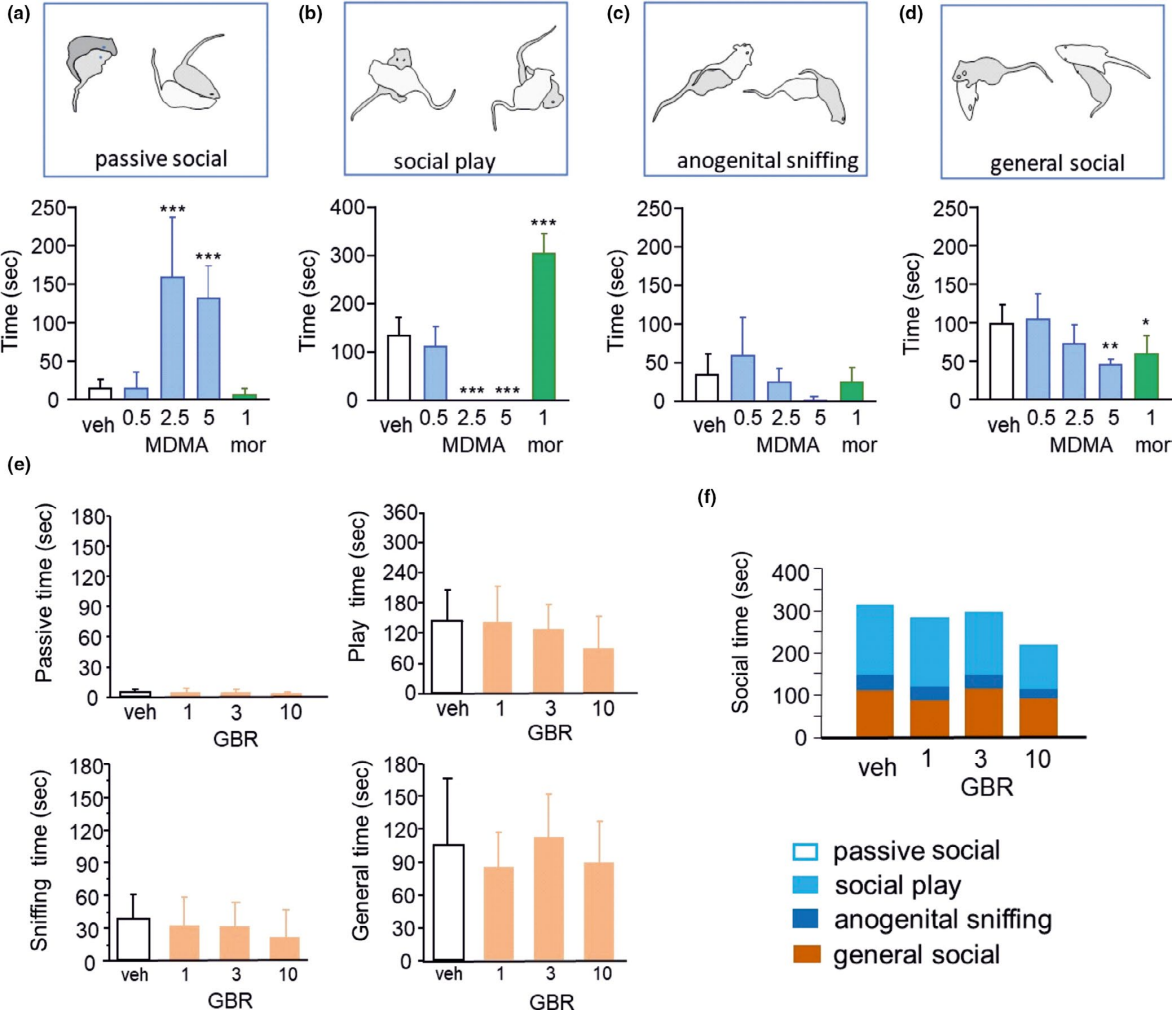
MDMA modulates social behavior in a distinct way from morphine. (a) MDMA increased the time spent in passive social behavior starting at a 2.5 mg/kg dose. (b) In contrast, MDMA reduced social time for other social interactions, such as social play, at 2.5 and 5 mg/kg doses. Morphine primarily increased social play, while passive social behavior remained intact in line with earlier findings. (c) Apart from a weak trend at the highest dose, MDMA or morphine failed to modulate anogenital sniffing. (d) General social behavior forms were affected by MDMA and morphine in a similar way, the effect of MDMA only being significant at the highest dose. Data represent means +/− SEM. *, **, and *** indicate *p* < 0.05, 0.01, 0.001, respectively, versus vehicle treatment; *n* = 8–12 rats/group. (e) GBR‐12909 applied alone was ineffective in changing all observed forms of social behavior. Upper left, time spent in passive social behavior; upper right, time spent in social play behavior; lower left, sniffing, and lower right, general social investigation time. Data represent the means + SD. (f) total social interaction time after treatment with GBR‐12909 shows some reduction at higher doses of GBR‐12909; *n* = 12 rats/group

**FIGURE 5:**
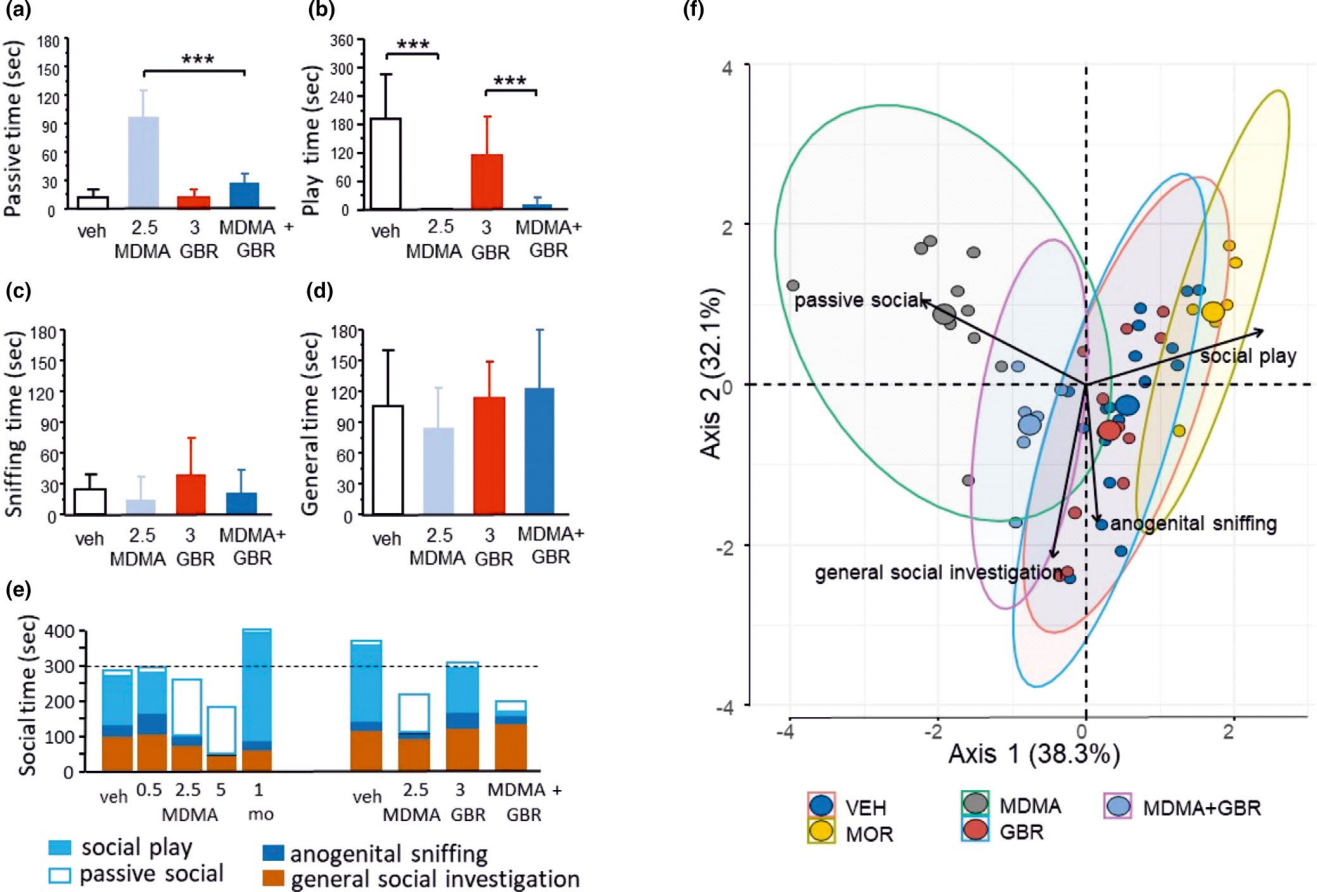
DAT inhibition restores specific elements of social behavior changed by MDMA. (a–d) The effects of MDMA, GBR‐12909 (GBR), and their combination on various aspects of social behavior during social interaction in rats. GBR‐12909 significantly reduced the 2.5 mg/kg MDMA‐induced time spent in passive social behavior. However, the inhibition of social play was not reversed by GBR‐12909. Anogenital sniffing and other forms of social behavior did not change. (e) Total time spent in social interaction after treatment with MDMA or morphine (left) and coadministration of MDMA with GBR‐12909 (right). The data are presented as the mean + SD. ***p < 0.001, Tukey's multiple comparisons test. (f) Principal component analysis of the forms of social behavior. Principal component analysis of the data shows the unique means by which MDMA changed social behavior compared to the means by which morphine changed social behavior. GBR‐12909 (when administered with MDMA) seemed to reverse this deviation and cause the behaviors to return to normal. The X‐axis shows the dimension that represents the largest variance (~38%) within the original four‐dimensional space; *n* = 12 rats/group

MDMA improved the passive social behavior of adult rats in a dose‐dependent manner, and this effect was significant at doses of 2.5 (*p* = 0.0003) and 5 mg/kg (*p* = 0.0021) (Figure 4a). While MDMA increased passive social behavior, it rather decreased other components of social behavior, such as social play (pinning) with significance observed at doses of 2.5 (*p *< 0.0001) and 5 mg/kg (*p *< 0.0001) (Figure 4b). Along with social play, general social investigation was also greatly reduced after MDMA treatment but only at the highest dose (*p *= 0.0119) (Figure 4d). Anogenital sniffing was not sensitive to either MDMA or morphine (Figure 4c). In contrast to the effects of MDMA, morphine treatment resulted in a completely different behavioral structure (Figures 4a–d and 5e). Consistent with earlier reports (Manduca et al., 2014), 1 mg/kg morphine significantly increased social play (*p *< 0.0001) but decreased general social investigation. In sharp contrast to MDMA, morphine did not change passive social behavior (*p* = 0.1702), emphasizing the specific modification of social behavior by different drugs. Apart from a weak trend to decrease social play at the highest dose of 10 mg/kg (*p* = 0.3770), the DAT inhibitor GBR‐12909 administered alone at doses of 1, 3, and 10 mg/kg failed to modulate elements of social behavior (Figure 4e), including passive social behavior (*p *= 0.6125), anogenital sniffing (*p *= 0.6783), and general social behavior (*p *= 0.6702). GBR‐12909 slightly reduced total social behavior (Figure 4f, *p* = 0.0792). Overall, DAT inhibition, applied alone, did not have significant influence on social behavior.

In a separate set of experiments, the modulatory effects of GBR‐12909 on the MDMA‐induced changes in social behavior were examined. The middle doses of the experiments, in which the drugs were applied alone, were chosen for both MDMA (2.5 mg/kg) and GBR‐12909 (3 mg/kg). Again, compared with the vehicle control, MDMA alone resulted in a significant increase in passive social behavior (*p *< 0.0001, Figure 5a), while GBR‐12909 alone did not alter passive social behavior (*p *= 0.999 vs. vehicle). When administered together with MDMA, GBR‐12909 significantly reduced the MDMA‐induced time spent engaged in passive social behavior (*p *< 0.0001 vs. MDMA) (Figure 5a). Interestingly, in contrast to the effect on passive social behavior, the clear inhibition of social play by MDMA (*p *= 0.0002 vs. control) could not be reversed by GBR‐12909 (*p* = 0.997 vs. MDMA), indicating that this effect of MDMA results from a DAT‐independent process of social behavior (Figure 5b). Anogenital sniffing was not significantly changed by the compounds when administered alone or in combination (*p *= 0.383, Figure 5c). General social investigation was also not significantly affected in this experiment (*p *= 0.549). In addition, compared to control, higher doses (2.5 and 5 mg/kg) of MDMA produced a nonsignificant trend to reduce the total duration of social behavior, while morphine increased the total time (*p* = 0.0031). Compared to the vehicle alone, in the combination experiments, 2.5 mg/kg MDMA reduced total social time which was not changed, the co‐application of GBR‐12909 (*p* = 0040 and *p* = 0.0013, respectively, Figure 5e). In addition, principal component analysis of the data further revealed unique changes in social behavior induced by MDMA and the specificity of DAT inhibition. In particular, the application of GBR‐12909 (when given with MDMA) shifted the deviation observed in Axis 1 toward the control values, suggesting a reversal of the MDMA‐induced changes in social behavior so that the DAT inhibition returns these behaviors to normal (Figure 5f). This is strongly supported by PERMANOVA results showing that the centroids of the MDMA/GBR‐12909 group differed from MDMA treatment (R2 = 0.408, F = 9.65, *p* < 0.001). PERMANOVA for all groups: (R2 = 0.67, F = 23.86, *p* < 0.001).

## 4 DISCUSSION

The role of transporters in the termination of transmitter action by uptake mechanism has been generally accepted (Torres et al., 2007). Transporters couple the inward movements of substrate to the movement of sodium ion down a concentration gradient and, in addition, they are able to function in reverse to carry transmitters resident in the cytoplasm into the extracellular space. These mechanisms are highly energy‐demanding and significantly contribute to the brain's energy usage on nonsignaling processes (Engl & Attwell, 2015). The contribution of monoamine transporters to extracellular levels of DA has been well established regarding release from cytoplasmic sources in response to amphetamines (Patriarchi et al., 2018; Sulzer et al., 1995; Zsilla et al., 2018). Under the conditions in these experiments (at 2 Hz frequency and in the presence of MDMA) the extracellular DA concentrations are the result of two types of release: the first is an extracellular [Ca^2+^]‐dependent one associated with APs, while the second is due to reversed operation of DAT in response to MDMA and is of cytoplasmic origin. The DA D2 antagonist sulpiride antagonized the inhibitory effects of MDMA on the evoked release of ACh at 2 Hz stimulation, while in the absence of MDMA (at the same frequency, 2 Hz) the evoked release of ACh was not affected by sulpiride (see also Figure 1d) indicating that the concomitantly released DA is not sufficient to affect ACh release. However, MDMA may also enhance stimulated DA release (see Figure 3b.) which could also inhibit ACh release in a sulpiride‐dependent manner. DA release from the cytoplasm is proven to be nonquantal in nature, in contrast to the release induced by neuronal firing, and is expected to manifest in behavioral changes. The structural similarity between MDMA and β‐PEA (O'Reilly et al., 1994) suggests that they act via the same system‐level target when inducing the release of DA from the cytoplasm. Given that β‐PEA is an endogenous molecule, this raises the possibility that it may potentially cause dopamine transporter reversal in "normal" brain processes. Furthermore, β‐PEA is implicated in psychiatric disorders (O'Reilly et al., 1991; 1994) and both MDMA and β‐PEA are thought to play a role in psychosis like in schizophrenia (O'Reilly et al., 1994). Supporting this view, PEA plasma levels are significantly higher in the schizophrenic patients compared with controls (O'Reilly et al., 1991) and this molecule is heterogeneously distributed in the brain along the evolution and found at the highest levels in the caudate putamen, olfactory tubercles, and NAc (Paterson et al.1990) and excreted in urine of patients with mental disorders (O'Reilly et al., 1994). Although, our data do not provide direct evidence for a role for endogenous B‐PEA in normal brain function as GBR‐12909 alone did not modify passive social behavior (Figure 4), it is possible that it could mediate other behaviors via analog communication which could become more pronounced with psychiatric illness. The β‐PEA‐induced increase in extracellular DA (Sotnikova et al., 2004), similar to that induced by amphetamine, is not affected by co‐perfusion of TTX (Westerink et al., 1987), confirming the action potential‐independent nature of this release (Zsilla et al., 2018). We found that DAT inhibition caused by either GBR‐12909 or, nonspecifically, by hypothermia (Bonnet et al., 1990) could inhibit the effects of MDMA or β‐PEA on the release of DA under resting conditions in the NAc. The activity of monoamine transporters is sensitive to temperature (Bonnet et al., 1990; Liang & Rutledge, 1982). The finding that hypothermia, used for the separation of vesicular from cytoplasmic transmitter release (Vizi, 1998; Vizi & Sperlágh, 1999) inhibited the basal release of DA in response to MDMA and β‐PEA (Figures 2 and 3) but potentiated the stimulation‐induced release of DA (first stimulation, Figure 2c), was a strong indication that the DAT was responsible for the effects. These results clearly indicate the direct cytoplasmic source of this basal release. GRB‐12909, cocaine and hypothermia, also decreased stimulated DA release in the presence of MDMA in the NAc (Figure 3b,c). The fact that cocaine, like GBR‐12909, a nonsubstrate blocker of DAT inhibited MDMA to release DA, is also an indication of the role of transporter. A similar observation was made by showing that cocaine blocked amphetamine‐induced DAT internalization (Wheeler et al., 2015). The pathological involvement of DAT has been shown in attention deficit hyperactivity disorder (Sakrikar et al., 2012) as well as in autism spectrum disorder (Bowton et al., 2014) and its reverse activity is associated with pathological conditions, like ischemia (Lakatos et al., 2020; Rossi et al., 2000).

The nonquantal release of DA by MDMA most likely targets D2 heteroreceptors located on ACh‐containing nerve terminals in the striatum, as verified by the finding that sulpiride, a D2 receptor antagonist, attenuated this effect (supported by Le Moine et al., 1990). The small attenuation of MDMA/PEA inhibition of stimulated ACh release following transporter blockade needs some explanation. Nonsynaptic communication opens channels between extrasynaptic release sites and remote receptors typically at extrasynaptic localization. This way of communication differs from synaptic transmission in a sense that it does not show such high efficiency, but several targets are affected at the same time. During electrical stimulation ACh is released anyway, so DA only need to modify it. At higher stimulation rate, a similar interaction was found by Riegert et al. (2008). We note that, at higher MDMA concentrations, an increase in the spontaneous ACh release could be observed in striatal slices (Fischer et al., 2000). On the other hand, besides cholinergic system, other mechanisms may also play a role in transmission between the dopaminergic activation and the prosocial behavior including the oxytocin‐containing neurons of hypothalamic nuclei (Otero‐Garcia et al., 2016). Moreover, nicotinic receptor antagonists could potentiate MDMA to enhance the peak of action‐potential‐mediated DA release DA release suggesting the potential involvement of nicotinic receptors in mediating the downstream effects following DA activation (Rizzo et al., 2018).

Compared with mice, rats have a more developed communication system and show a wider set of social behavioral elements with easily identifiable mutual social play behavior (Brust et al., 2015), thus rats were used for the in vivo experiments. In this study we have shown that the dose of MDMA that increased adjacent lying, the most common passive social behavior (Kamilar‐Britt & Bedi, 2015), reduced social play at the same time. This result indicates that MDMA interferes with social behavior in a bidirectional way; one element of social behavior is enhanced while another element is reduced. Traditionally, modulation of social behavior by MDMA has been associated with serotonergic neurotransmission (Homberg et al., 2007; Morley et al., 2005) and the oxytocin/vasopressin systems (Ramos et al., 2016). MDMA‐induced serotonin transporter reversal in the NAc increases prosocial behavior via interactions with 5‐HT1b receptors on striatal glutamatergic terminals that synapse onto medium spiny neurons (Dölen et al., 2013; Heifets et al., 2019). Data from the literature indirectly suggest that DA in the mesolimbic pathway may be involved in the regulation of social behavior by MDMA (Vanderschuren et al., 2008); however, little is known about the role of DA in social neural networks and the mechanism by which DA mediates the central action of MDMA, despite the clear connection between DA and reward‐driven behavior. The inhibitory effect of GBR‐12909 on MDMA‐induced DA release indicates that DATs and thereby the cytoplasmic release of DA are involved in the modulation of passive social behavior produced by MDMA. In contrast, GBR‐12909 was unable to reverse the suppressive effect of MDMA on social play (Figure 5b), emphasizing the specificity of the effects of cytoplasmic DA on social behavior. These findings suggest that nonquantal DA release most likely plays a role in a distinct set of social behaviors induced by MDMA, while other changes in social behavior following MDMA are mediated via different mechanisms. Indeed, other psychostimulant compounds, namely, methylphenidate (Vanderschuren et al., 2008) and cocaine, inhibitors of monoamine transporters, also reduce social play. In support of this hypothesis, in contrast to GBR‐12909, the selective NE reuptake inhibitor atomoxetine reduced social play (Vanderschuren et al., 2008). This finding may indicate that while the cytosolic/nonquantal release of DA plays a specific role in the regulation of passive social behavior by MDMA, it does not play a regulatory role in the suppression of other characteristic social behaviors, such as social play where other transmitter systems regulate the behavior.

**FIGURE 6:**
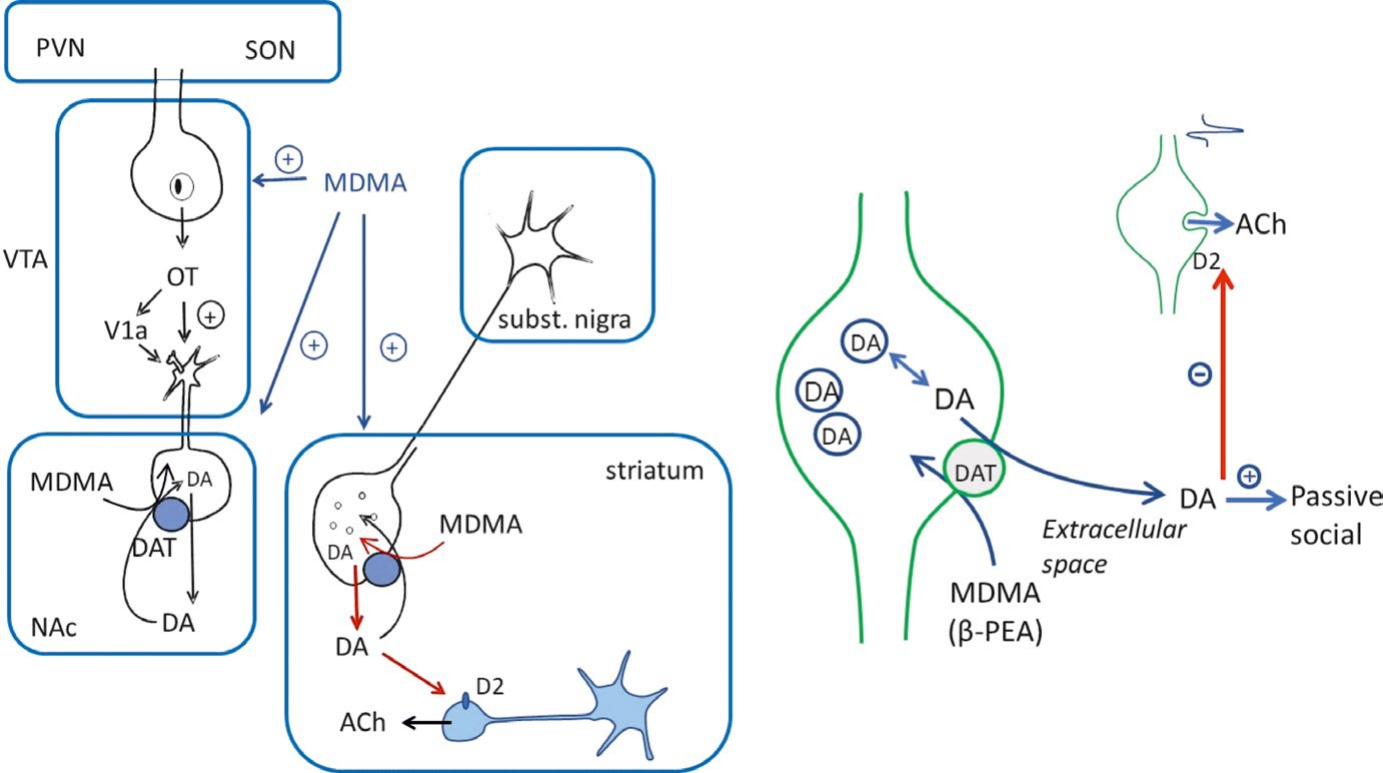
Schematic drawings of the background neural mini‐network underlying the higher‐level effects of MDMA. The inset shows the membrane‐ and system‐level effects of MDMA on local DA and ACh release. By modifying the VTA pathway, MDMA can influence DA release in the NAc in vivo. There are direct effects of MDMA on striatal and accumbal DA release that can be separately studied in slice preparations. The reverse function of DAT activity induced by the inward transport of MDMA (substrate of DAT) from the extracellular environment induces the release of DA from the cytoplasm. GBR‐12909, which is a selective inhibitor of DAT, and hypothermia (17°C) prevents MDMA from inducing the nonquantal release of DA. It should be noted that the forward and reverse transport mechanisms are mediated by the same transporter. The extracellular DA, elevated by MDMA, contributes to a specific behavioral effect of MDMA, the passive social behavior. The indirect effect of MDMA on ACh release in the striatum is shown. NAc: nucleus accumbens, VTA: ventral tegmental area, PVN: paraventricular nucleus, SON: supraoptic nucleus, OT: oxytocin

On a broader scale, oxytocin‐containing neurons from the hypothalamic paraventricular (PVN) and supraoptic nuclei (SON) send projections to the VTA and NAc (Otero‐Garcia et al., 2016) and have been implicated in social reward‐driven behaviors (Love, 2014). Oxytocin receptors are expressed on dopaminergic cell bodies in the VTA but not on dopaminergic terminals in the NAc (Peris et al., 2017). Oxytocinergic fibers in the VTA could activate DA in the NAc (Melis et al., 2007; Tang et al., 2014) via receptors expressed on cell bodies of dopaminergic neurons (Figure 6). Therefore, our finding that MDMA released DA in NAc slices, albeit in the presence of a monoamine oxidase inhibitor, suggests that there is a direct effect of MDMA on local DA release (Figure 6). MDMA increases oxytocin levels both in vitro (Forsling et al., 2002) and in vivo (Thompson et al., 2007), suggesting the role of an indirect link between oxytocin and MDMA‐induced DA release.

In the central nervous system, relatively few monoaminergic terminals, including nigrostriatal and mesolimbic dopaminergic terminals, form synaptic contacts (Descarries & Mechawar, 2000), indicating that most functional interactions between neurons could be considered mostly nonsynaptic (Vizi, 1984). DA released from nonsynaptic boutons directly signals through a large territory due to the anatomy of dopaminergic axonal arborizations (Matsuda et al., 2009). Brain extracellular space forms pathways for nonsynaptic diffusion in neurochemical signaling and drug delivery (Rice & Nicholson, 1991). Cholinergic terminals contain D2 receptors, and these receptors could be reached easily by diffusion by extracellular DA. As shown in our experiments, the cytoplasmic/nonquantal release of DA induced by MDMA in the striatum can affect the axonal stimulation‐induced release of ACh (Figure 2d) from cholinergic interneurons without synaptic contact (Descarries et al., 1997) via the activation of D2 receptors (Figure 2e). The primary nonsynaptic localization of DAT in the mesocorticolimbic area (~95%) (Nirenberg et al., 1997), the predominance of extrasynaptic D2 receptors (Sesack et al., 1994) and the morphological findings that nigrostriatal boutons form relatively few synaptic contacts (Descarries et al., 1996) further support our conclusion that in the brain, neuronal firing‐dependent synaptic activity is not the sole driver of behavior. In the nonsynaptic modulation of chemical transmission (Vizi, 1984), neuronal targets do not receive synaptic inputs but express high‐affinity receptors and transporters that accept messages arriving as low concentrations of transmitters (three orders of magnitude lower than those in the synapse) or drugs taken by patients and accordingly, they are capable of exerting long‐lasting tonic effects (Vizi et al., 2010).

While synapses between neurons include the precise flow of digital information at millisecond‐level speeds, neurotransmitters released into the extracellular space from nonsynaptic terminals operate as slow waves of neurotransmitter concentrations around distant neurons and exert long‐lasting effects. To complicate this view, rapid dopamine transients can be detected using fast scan cyclic voltammetry in the NAc due to reward expectation (Phillips et al., 2003) indicating that DA does not just signal on slow time frames as slow waves. The synchronization of spiking activity in neuronal networks is a crucial process that enables transmission to be precise and capable of driving behavioral responses (Buzsáki & Draguhn, 2004).

The present results reveal a particular case in which analog (nonquantal) activity in the brain could contribute to social behavior. In particular, we report that DA could be released from the cytoplasm of axon terminals in response to β‐PEA or MDMA via the reverse function of DATs, indicating that cytoplasmic, nonvesicular DA release could convey the same information as dopaminergic neuron firing or at least be able to help increase and/or maintain DA concentrations in extracellular space by series of presynaptic spikes. MDMA‐evoked DA release at rest is the overwhelming transmitter‐level action both in the striatum and in the NAc to induce changes in local circuits. Although the contribution of enhanced stimulation‐evoked DA release to this phenomenon cannot be excluded in a minor extent, the vast majority of the effect should come from resting release, which is contributed by the nonquantal transporter reversal effect. Moreover, even if vesicular release adds to the released DA, it reaches the target via extrasynaptic routes where DA of nonsynaptic origin is most likely the dominant contributor. Our findings, that the selective inhibition of DAT prevents MDMA to release DA into the extracellular space nonquantally and to tonically inhibit ACh release from nonsynaptic cholinergic terminals and in in vivo experiments GBR‐12909 is able to change behavior characteristic of MDMA, provide convincing evidence that in addition to digital transmission, analog communication is also involved in brain function. Following MDMA applications DA plays a role in MDMA ability to modify an aspect of prosocial behavior altering the current view on the cellular mechanisms used by MDMA in the brain.

## CONFLICT OF INTEREST

The authors declare no conflict of interest.

## AUTHOR CONTRIBUTIONS

E.S.V, B.L., Zs.N. B.S., and V.R. conceived the project, worked out the concept and design of the work, and analyzed the data. E.S.V and B.L. wrote the manuscript. K.K. and R.K. participated in acquisition, analysis, and interpretation of data for the work. E.S.V, B.L., Zs.N. B.S., and V.R revised the work critically for important intellectual content.

## Data Availability

All relevant data are within the article.

## References

[phy215088-bib-0001] Alcaro, A. , Huber, R. , & Panksepp, J. (2007). Behavioral functions of the mesolimbic dopaminergic system: An affective neuroethological perspective. Brain Research Reviews, 56, 283–321. 10.1016/j.brainresrev.2007.07.014 17905440 PMC2238694

[phy215088-bib-0002] Ando, R. D. , Benko, A. , Ferrington, L. , Kirilly, E. , Kelly, P. A. , & Bagdy, G. (2006). Partial lesion of the serotonergic system by a single dose of MDMA results in behavioural disinhibition and enhances acute MDMA‐induced social behaviour on the social interaction test. Neuropharmacology, 50, 884–896. 10.1016/j.neuropharm.2005.12.010 16472832

[phy215088-bib-0003] Arias‐Carrión, O. , Stamelou, M. , Murillo‐Rodríguez, E. , Menéndez‐González, M. , & Pöppel, E. (2010). Dopaminergic reward system: A short integrative review. International Archives of Medicine, 3, 24. 10.1186/1755-7682-3-24 20925949 PMC2958859

[phy215088-bib-0004] Bartholini, G. , Stadler, H. , Ciria, M. G. , & Lloyd, K. G. (1976). The use of the push‐pull cannula to estimate the dynamics of acetylcholine and catecholamines within various brain areas. Neuropharmacology, 15, 515–519. 10.1016/0028-3908(76)90101-5 980227

[phy215088-bib-0005] Bonnet, J. J. , Benmansour, S. , Costentin, J. , Parker, E. M. , & Cubeddu, L. X. (1990). Thermodynamic analyses of the binding of substrates and uptake inhibitors on the neuronal carrier of dopamine labeled with [^3^H]GBR 12783 or [^3^H]mazindol. Journal of Pharmacology and Experimental Therapeutics, 253, 1206–1214 2141637

[phy215088-bib-0006] Bowton, E. , Saunders, C. , Reddy, I. A. , Campbell, N. G. , Hamilton, P. J. , Henry, L. K. , Coon, H. , Sakrikar, D. , Veenstra‐VanderWeele, J. M. , Blakely, R. D. , Sutcliffe, J. , Matthies, H. J. , Erreger, K. , & Galli, A. (2014). SLC6A3 coding variant Ala559Val found in two autism probands alters dopamine transporter function and trafficking. Translational Psychiatry, 4, e464. 10.1038/tp.2014.90 25313507 PMC4350523

[phy215088-bib-0007] Brooks, J. M. , Sarter, M. , & Bruno, J. P. (2007). D2‐like receptors in nucleus accumbens negatively modulate acetylcholine release in prefrontal cortex. Neuropharmacology, 53, 455–463. 17681559 10.1016/j.neuropharm.2007.06.006 PMC2000917

[phy215088-bib-0008] Brust, V. , Schindler, P. M. , & Lewejohann, L. (2015). Lifetime development of behavioural phenotype in the house mouse (Mus musculus). Frontiers in Zoology, 12(Suppl 1), S17. 10.1186/1742-9994-12-S1-S17 26816516 PMC4722345

[phy215088-bib-0009] Buzsaki, G. , & Draguhn, A. (2004). Neuronal oscillations in cortical networks. Science, 304, 1926–1929. 10.1126/science.1099745 15218136

[phy215088-bib-0010] Chi, L. , & Reith, M. E. (2003). Substrate‐induced trafficking of the dopamine transporter in heterologously expressing cells and in rat striatal synaptosomal preparations. Journal of Pharmacology and Experimental Therapeutics, 307, 729–736. 10.1124/jpet.103.055095 12975490

[phy215088-bib-0011] Descarries, L. , Gisiger, V. , & Steriade, M. (1997). Diffuse transmission by acetylcholine in the CNS. Progress in Neurobiology, 53, 603–625. 10.1016/S0301-0082(97)00050-6 9421837

[phy215088-bib-0012] Descarries, L. , & Mechawar, N. (2000). Ultrastructural evidence for diffuse transmission by monoamine and acetylcholine neurons of the central nervous system. Progress in Brain Research, 125, 27–47. 11098652 10.1016/S0079-6123(00)25005-X

[phy215088-bib-0013] Descarries, L. , Watkins, K. C. , Garcia, S. , Bosler, O. , & Doucet, G. (1996). Dual character, asynaptic and synaptic, of the dopamine innervation in adult rat neostriatum: A quantitative autoradiographic and immunocytochemical analysis. The Journal of Comparative Neurology, 375, 167–186. 8915824 10.1002/(SICI)1096-9861(19961111)375:2<167::AID-CNE1>3.0.CO;2-0

[phy215088-bib-0014] Dölen, G. , Darvishzadeh, A. , Huang, K. W. , & Malenka, R. C. (2013). Social reward requires coordinated activity of nucleus accumbens oxytocin and serotonin. Nature, 501, 179–184. 10.1038/nature12518 24025838 PMC4091761

[phy215088-bib-0015] Engl, E. , & Attwell, D. (2015). Non‐signalling energy use in the brain. Journal of Physiology, 593, 3417–3429. 10.1113/jphysiol.2014.282517 PMC4560575 25639777

[phy215088-bib-0016] Fischer, H. S. , Zernig, G. , Schatz, D. S. , Humpel, C. , & Saria, A. (2000). MDMA ('ecstasy') enhances basal acetylcholine release in brain slices of the rat striatum. European Journal of Neuroscience, 12, 1385–1390. 10.1046/j.1460-9568.2000.00004.x 10762366

[phy215088-bib-0017] Forsling, M. L. , Fallon, J. K. , Shah, D. , Tilbrook, G. S. , Cowan, D. A. , Kicman, A. T. , & Hutt, A. J. (2002). The effect of 3,4‐methylenedioxymethamphetamine (MDMA, ‘ecstasy’) and its metabolites on neurohypophysial hormone release from the isolated rat hypothalamus. British Journal of Pharmacology, 135, 649–656. 10.1038/sj.bjp.0704502 11834612 PMC1573171

[phy215088-bib-0018] Giros, B. , Jaber, M. , Jones, S. R. , Wightman, R. M. , & Caron, M. G. (1996). Hyperlocomotion and indifference to cocaine and amphetamine in mice lacking the dopamine transporter. Nature, 379, 606–612. 10.1038/379606a0 8628395

[phy215088-bib-0019] Gudelsky, G. A. , & Yamamoto, B. K. (2008). Actions of 3,4‐methylenedioxymethamphetamine (MDMA) on cerebral dopaminergic, serotonergic and cholinergic neurons. Pharmacology, Biochemistry and Behavior, 90, 198–207. 10.1016/j.pbb.2007.10.003 PMC2505334 18035407

[phy215088-bib-0020] Heifets, B. D. , Salgado, J. S. , Taylor, M. D. , Hoerbelt, P. , Cardozo Pinto, D. F. , Steinberg, E. E. , Walsh, J. J. , Sze, J. Y. , & Malenka, R. C. (2019). Distinct neural mechanisms for the prosocial and rewarding properties of MDMA. Science Translational Medicine, 11(522), eaaw6435. 10.1126/scitranslmed.aaw6435 31826983 PMC7123941

[phy215088-bib-0021] Homberg, J. R. , Schiepers, O. J. , Schoffelmeer, A. N. , Cuppen, E. , & Vanderschuren, L. J. (2007). Acute and constitutive increases in central serotonin levels reduce social play behaviour in peri‐adolescent rats. Psychopharmacology (Berl), 195, 175–182. 10.1007/s00213-007-0895-8 17661017 PMC2048539

[phy215088-bib-0022] Iravani, M. M. , Asari, D. , Patel, J. , Wieczorek, W. J. , & Kruk, Z. L. (2000). Direct effects of 3,4‐methylenedioxymethamphetamine (MDMA) on serotonin or dopamine release and uptake in the caudate putamen, nucleus accumbens, substantia nigra pars reticulata, and the dorsal raphé nucleus slices. Synapse (New York, N. Y.), 36, 275–285. 10.1002/(SICI)1098-2396(20000615)36:4<275::AID-SYN4>3.0.CO;2-# 10819905

[phy215088-bib-0023] Janssen, P. A. J. , Leysen, J. E. , Megens, A. A. H. P. , & Awouters, F. H. L. (1999). Does phenylethylamine act as an endogenous amphetamine in some patients? International Journal of Neuropsychopharmacology, 2, 229–240. 10.1017/S1461145799001522 11281991

[phy215088-bib-0024] Jones, S. R. , Gainetdinov, R. R. , Wightman, R. M. , & Caron, M. G. (1998). Mechanisms of amphetamine action revealed in mice lacking the dopamine transporter. Journal of Neuroscience, 18, 1979–1986. 10.1523/JNEUROSCI.18-06-01979.1998 9482784 PMC6792915

[phy215088-bib-0025] Kamilar‐Britt, P. , & Bedi, G. (2015). The prosocial effects of 3,4‐methylenedioxymethamphetamine (MDMA): Controlled studies in humans and laboratory animals. Neuroscience and Biobehavioral Reviews, 57, 433–446. 10.1016/j.neubiorev.2015.08.016 26408071 PMC4678620

[phy215088-bib-0026] Lakatos, M. , Baranyi, M. , Erőss, L. , Nardai, S. , Török, T. L. , Sperlágh, B. , & Vizi, E. S. (2020). Roles played by the Na(^+^)/Ca(^2+^) exchanger and hypothermia in the prevention of ischemia‐induced carrier‐mediated efflux of catecholamines into the extracellular space: Implications for stroke therapy. Neurochemical Research, 45, 16–33. 10.1007/s11064-019-02842-0 31346893 PMC6942591

[phy215088-bib-0027] Le Moine, C. , Tison, F. , & Bloch, B. (1990). D2 dopamine receptor gene expression by cholinergic neurons in the rat striatum. Neuroscience Letters, 117, 248–252. 10.1016/0304-3940(90)90671-U 2094817

[phy215088-bib-0028] Lendvai, B. , Sándor, N. T. , & Sándor, A. (1993). Influence of selective opiate antagonists on striatal acetylcholine and dopamine release. Acta Physiologica Hungarica, 81, 19–28. 8178651

[phy215088-bib-0029] Liang, N. Y. , & Rutledge, C. O. (1982). Comparison of the release of [^3^H]dopamine from isolated corpus striatum by amphetamine, fenfluramine and unlabelled dopamine. Biochemical Pharmacology, 31, 983–992. 10.1016/0006-2952(82)90332-X 7082379

[phy215088-bib-0030] Love, T. M. (2014). Oxytocin, motivation and the role of dopamine. Pharmacology, Biochemistry and Behavior, 119, 49–60. 10.1016/j.pbb.2013.06.011 PMC3877159 23850525

[phy215088-bib-0031] Manduca, A. , Campolongo, P. , Palmery, M. , Vanderschuren, L. J. , Cuomo, V. , & Trezza, V. (2014). Social play behavior, ultrasonic vocalizations and their modulation by morphine and amphetamine in Wistar and Sprague‐Dawley rats. Psychopharmacology (Berl), 231, 1661–1673. 10.1007/s00213-013-3337-9 24221828

[phy215088-bib-0032] Matsuda, W. , Furuta, T. , Nakamura, K. C. , Hioki, H. , Fujiyama, F. , Arai, R. , & Kaneko, T. (2009). Single nigrostriatal dopaminergic neurons form widely spread and highly dense axonal arborizations in the neostriatum. Journal of Neuroscience, 29, 444–453. 10.1523/JNEUROSCI.4029-08.2009 19144844 PMC6664950

[phy215088-bib-0033] McArdle, B. H. , & Anderson, M. J. (2001). Fitting multivariate models to community data: A comment on distance‐based redundancy analysis. Ecology, 82, 290–297.

[phy215088-bib-0034] Melis, M. R. , Melis, T. , Cocco, C. , Succu, S. , Sanna, F. , Pillolla, G. , Boi, A. , Ferri, G. L. , & Argiolas, A. (2007). Oxytocin injected into the ventral tegmental area induces penile erection and increases extracellular dopamine in the nucleus accumbens and paraventricular nucleus of the hypothalamus of male rats. European Journal of Neuroscience, 26, 1026–1035. 10.1111/j.1460-9568.2007.05721.x 17672853

[phy215088-bib-0035] Milusheva, E. , Baranyi, M. , Kittel, A. , Fekete, A. , Zelles, T. , Vizi, E. S. , & Sperlágh, B. (2008). Modulation of dopaminergic neurotransmission in rat striatum upon in vitro and in vivo diclofenac treatment. Journal of Neurochemistry, 105, 360–368. 10.1111/j.1471-4159.2007.05141.x 18036194 PMC2324205

[phy215088-bib-0036] Morley, K. C. , Arnold, J. C. , & McGregor, I. S. (2005). Serotonin (1A) receptor involvement in acute 3,4‐methylenedioxymethamphetamine (MDMA) facilitation of social interaction in the rat. Progress in Neuro‐Psychopharmacology and Biological Psychiatry, 29, 648–657. 15908091 10.1016/j.pnpbp.2005.04.009

[phy215088-bib-0037] Moss, J. , & Bolam, J. P. (2008). A dopaminergic axon lattice in the striatum and its relationship with cortical and thalamic terminals. Journal of Neuroscience, 28, 11221–11230. 10.1523/JNEUROSCI.2780-08.2008 18971464 PMC6671499

[phy215088-bib-0038] (2018). Multivariate analysis in the pharmaceutical industry (pp. 26–27). 10.1016/C2016-0-00555-7

[phy215088-bib-0039] Nirenberg, M. J. , Chan, J. , Vaughan, R. A. , Uhl, G. R. , Kuhar, M. J. , & Pickel, V. M. (1997). Immunogold localization of the dopamine transporter: An ultrastructural study of the rat ventral tegmental area. Journal of Neuroscience, 17, 5255–5262. 10.1523/JNEUROSCI.17-14-05255.1997 9204909 PMC6793826

[phy215088-bib-0040] O'Reilly, R. L. , & Davis, B. A. (1994). Phenylethylamine and schizophrenia. Progress in Neuro‐Psychopharmacology and Biological Psychiatry, 18, 63–75. 10.1016/0278-5846(94)90024-8 7906896

[phy215088-bib-0041] O'Reilly, R. , Davis, B. A. , Durden, D. A. , Thorpe, L. , Machnee, H. , & Boulton, A. A. (1991). Plasma phenylethylamine in schizophrenic patients. Biological Psychiatry, 30, 145–150. 1912106 10.1016/0006-3223(91)90168-l

[phy215088-bib-0042] Otero‐Garcia, M. , Agustín‐Pavón, C. , Lanuza, E. , & Martínez‐García, F. (2016). Distribution of oxytocin and co‐localization with arginine vasopressin in the brain of mice. Brain Struct Funct, 221, 3445–3473. 10.1007/s00429-015-1111-y 26388166

[phy215088-bib-0043] Paterson, I. A. , Juorio, A. V. , & Boulton, A. A. (1990). 2‐Phenylethylamine: A modulator of catecholamine transmission in the mammalian central nervous system? Journal of Neurochemistry, 55, 1827–1837. 10.1111/j.1471-4159.1990.tb05764.x 2172461

[phy215088-bib-0044] Patriarchi, T. , Cho, J. R. , Merten, K. , Howe, M. W. , Marley, A. , Xiong, W. H. , Folk, R. W. , Broussard, G. J. , Liang, R. , Jang, M. J. , Zhong, H. , Dombeck, D. , von Zastrow, M. , Nimmerjahn, A. , Gradinaru, V. , Williams, J. T. , & Tian, L. (2018). Ultrafast neuronal imaging of dopamine dynamics with designed genetically encoded sensors. Science, 360, eaat4422. 10.1126/science.aat4422 29853555 PMC6287765

[phy215088-bib-0045] Peris, J. , MacFadyen, K. , Smith, J. A. , de Kloet, A. D. , Wang, L. , & Krause, E. G. (2017). Oxytocin receptors are expressed on dopamine and glutamate neurons in the mouse ventral tegmental area that project to nucleus accumbens and other mesolimbic targets. The Journal of Comparative Neurology, 525, 1094–1108. 10.1002/cne.24116 27615433 PMC6483090

[phy215088-bib-0046] Phillips, P. E. , Stuber, G. D. , Heien, M. L. , Wightman, R. M. , & Carelli, R. M. (2003). Subsecond dopamine release promotes cocaine seeking. Nature, 422, 614–618. 10.1038/nature01476 12687000

[phy215088-bib-0047] Pitts, E. G. , Minerva, A. R. , Chandler, E. B. , Kohn, J. N. , Logun, M. T. , Sulima, A. , Rice, K. C. , & Howell, L. L. (2017). 3,4‐methylenedioxymethamphetamine increases affiliative behaviors in squirrel monkeys in a serotonin 2A receptor‐dependent manner. Neuropsychopharmacology, 42, 1962–1971. 10.1038/npp.2017.80 28425496 PMC5561347

[phy215088-bib-0048] R Core Team (2013). R: A language and environment for statistical computing. R Foundation for Statistical Computing. http://www.R‐project.org/.

[phy215088-bib-0049] Raiteri, L. , & Raiteri, M. (2015). Multiple functions of neuronal plasma membrane neurotransmitter transporters. Progress in Neurobiology, 134, 1–16. 10.1016/j.pneurobio.2015.08.002 26300320

[phy215088-bib-0050] Ramos, L. , Hicks, C. , Caminer, A. , Couto, K. , Narlawar, R. , Kassiou, M. , & McGregor, I. S. (2016). MDMA ('Ecstasy'), oxytocin and vasopressin modulate social preference in rats: A role for handling and oxytocin receptors. Pharmacology, Biochemistry and Behavior, 150–151, 115–123. 10.1016/j.pbb.2016.10.002 27725273

[phy215088-bib-0051] Rice, M. E. , & Nicholson, C. (1991). Diffusion characteristics and extracellular volume fraction during normoxia and hypoxia in slices of rat neostriatum. Journal of Neurophysiology, 65, 264–276. 10.1152/jn.1991.65.2.264 2016641

[phy215088-bib-0052] Riegert, C. , Wedekind, F. , Hamida, S. B. , Rutz, S. , Rothmaier, A. K. , Jones, B. C. , Cassel, J. C. , & Jackisch, R. (2008). Effects of ethanol and 3,4‐methylenedioxymethamphetamine (MDMA) alone or in combination on spontaneous and evoked overflow of dopamine, serotonin and acetylcholine in striatal slices of the rat brain. International Journal of Neuropsychopharmacology, 11, 743–763. 10.1017/S1461145708008481 18248690

[phy215088-bib-0053] Rizzo, F. R. , Federici, M. , & Mercuri, N. B. (2018). 3,4‐Methylenedioxymethamphetamine (MDMA) alters synaptic dopamine release in the dorsal striatum through nicotinic receptors and DAT inhibition. Neuroscience, 377, 69–76. 29510210 10.1016/j.neuroscience.2018.02.037

[phy215088-bib-0054] Rossi, D. J. , Oshima, T. , & Attwell, D. (2000). Glutamate release in severe brain ischaemia is mainly by reversed uptake. Nature, 403, 316–321. 10.1038/35002090 10659851

[phy215088-bib-0055] Sakrikar, D. , Mazei‐Robison, M. S. , Mergy, M. A. , Richtand, N. W. , Han, Q. , Hamilton, P. J. , Bowton, E. , Galli, A. , Veenstra‐Vanderweele, J. , Gill, M. , & Blakely, R. D. (2012). Attention deficit/hyperactivity disorder‐derived coding variation in the dopamine transporter disrupts microdomain targeting and trafficking regulation. Journal of Neuroscience, 32, 5385–5397. 10.1523/JNEUROSCI.6033-11.2012 22514303 PMC3342037

[phy215088-bib-0056] Saunders, C. , Ferrer, J. V. , Shi, L. , Chen, J. , Merrill, G. , Lamb, M. E. , Leeb‐Lundberg, L. M. , Carvelli, L. , Javitch, J. A. , & Galli, A. (2000). Amphetamine‐induced loss of human dopamine transporter activity: An internalization‐dependent and cocaine‐sensitive mechanism. Proceedings of the National Academy of Sciences, 97, 6850–6855. 10.1073/pnas.110035297 PMC18764 10823899

[phy215088-bib-0057] Sesack, S. R. , Aoki, C. , & Pickel, V. M. (1994). Ultrastructural localization of D2 receptor‐like immunoreactivity in midbrain dopamine neurons and their striatal targets. Journal of Neuroscience, 14, 88–106. 7904306 10.1523/JNEUROSCI.14-01-00088.1994 PMC6576858

[phy215088-bib-0058] Sesack, S. R. , & Pickel, V. M. (1990). In the rat medial nucleus accumbens, hippocampal and catecholaminergic terminals converge on spiny neurons and are in apposition to each other. Brain Research, 527, 266–279. 10.1016/0006-8993(90)91146-8 1701338

[phy215088-bib-0059] Smith, Y. , & Bolam, J. P. (1990). The output neurones and the dopaminergic neurones of the substantia nigra receive a GABA‐containing input from the globus pallidus in the rat. Journal of Comparative Neurology, 296, 47–64. 10.1002/cne.902960105 1694189

[phy215088-bib-0060] Sitte, H. H. , Huck, S. , Reither, H. , Boehm, S. , Singer, E. A. , & Pifl, C. (1998). Carrier‐mediated release, transport rates, and charge transfer induced by amphetamine, tyramine, and dopamine in mammalian cells transfected with the human dopamine transporter. Journal of Neurochemistry, 71, 1289–1297. 10.1046/j.1471-4159.1998.71031289.x 9721755

[phy215088-bib-0061] Sotnikova, T. D. , Budygin, E. A. , Jones, S. R. , Dykstra, L. A. , Caron, M. G. , & Gainetdinov, R. R. (2004). Dopamine transporter‐dependent and ‐independent actions of trace amine beta‐phenylethylamine. Journal of Neurochemistry, 91, 362–373. 10.1111/j.1471-4159.2004.02721.x 15447669

[phy215088-bib-0062] Sulzer, D. , Chen, T. K. , Lau, Y. Y. , Kristensen, H. , Rayport, S. , & Ewing, A. (1995). Amphetamine redistributes dopamine from synaptic vesicles to the cytosol and promotes reverse transport. Journal of Neuroscience, 15, 4102–4108. 10.1523/JNEUROSCI.15-05-04102.1995 7751968 PMC6578196

[phy215088-bib-0063] Sulzer, D. , Maidment, N. T. , & Rayport, S. (1993). Amphetamine and other weak bases act to promote reverse transport of dopamine in ventral midbrain neurons. Journal of Neurochemistry, 60, 527–535. 10.1111/j.1471-4159.1993.tb03181.x 8419534

[phy215088-bib-0064] Tang, Y. , Chen, Z. , Tao, H. , Li, C. , Zhang, X. , Tang, A. , & Liu, Y. (2014). Oxytocin activation of neurons in ventral tegmental area and interfascicular nucleus of mouse midbrain. Neuropharmacology, 77, 277–284. 10.1016/j.neuropharm.2013.10.004 24148809

[phy215088-bib-0065] Thompson, M. R. , Callaghan, P. D. , Hunt, G. E. , Cornish, J. L. , & McGregor, I. S. (2007). A role for oxytocin and 5‐HT(1A) receptors in the prosocial effects of 3,4 methylenedioxymethamphetamine ("ecstasy"). Neuroscience, 146, 509–514. 10.1016/j.neuroscience.2007.02.032 17383105

[phy215088-bib-0066] Torres, G. E. , & Amara, S. G. (2007). Glutamate and monoamine transporters: New visions of form and function. Current Opinion in Neurobiology, 17, 304–312. 10.1016/j.conb.2007.05.002 17509873

[phy215088-bib-0067] Vanderschuren, L. J. , Achterberg, E. J. , & Trezza, V. (2016). The neurobiology of social play and its rewarding value in rats. Neuroscience and Biobehavioral Reviews, 70, 86–105. 27587003 10.1016/j.neubiorev.2016.07.025 PMC5074863

[phy215088-bib-0068] Vanderschuren, L. J. , Trezza, V. , Griffioen‐Roose, S. , Schiepers, O. J. , Van Leeuwen, N. , De Vries, T. J. , & Schoffelmeer, A. N. (2008). Methylphenidate Disrupts Social Play Behavior in Adolescent Rats. Neuropsychopharmacology, 33, 2946–2956. 10.1038/npp.2008.10 18305462 PMC2580804

[phy215088-bib-0069] Venables, W. N. , & Ripley, B. D. (2002). Modern Applied Statistics with S, 4th ed. Springer. http://www.stats.ox.ac.uk/pub/MASS4.

[phy215088-bib-0070] Vizi, E. S. (1984). Non‐synaptic interactions between neurons: Modulation of neurochemical transmission. Wiley.

[phy215088-bib-0071] Vizi, E. S. (1998). Different temperature dependence of carrier‐mediated (cytoplasmic) and stimulus‐evoked (exocytotic) release of transmitter: A simple method to separate the two types of release. Neurochemistry International, 33, 359–366. 10.1016/S0197-0186(98)00040-0 9840227

[phy215088-bib-0072] Vizi, E. S. , Fekete, A. , Karoly, R. , & Mike, A. (2010). Non‐synaptic receptors and transporters involved in brain functions and targets of drug treatment. British Journal of Pharmacology, 160, 785–809. 10.1111/j.1476-5381.2009.00624.x 20136842 PMC2935987

[phy215088-bib-0073] Vizi, E. S. , Hársing, L. G. Jr , & Knoll, J. (1977). Presynaptic inhibition leading to disinhibition of acetylcholine release from interneurons of the caudate nucleus: Effects of dopamine, beta‐endorphin and D‐Ala2‐Pro5 enkephalinamide. Neuroscience, 2, 953–961.

[phy215088-bib-0074] Vizi, E. S. , & Sperlágh, B. (1999). Separation of carrier mediated and vesicular release of GABA from rat brain slices. Neurochemistry International, 34, 407–413. 10.1016/S0197-0186(99)00047-9 10397369

[phy215088-bib-0075] Westerink, B. H. , Tuntler, J. , Damsma, G. , Rollema, H. , & de Vries, J. B. (1987). The use of tetrodotoxin for the characterization of drug‐enhanced dopamine release in conscious rats studied by brain dialysis. Naunyn‐Schmiedeberg's Archives of Pharmacology, 336, 502–507. 10.1007/BF00169306 3501841

[phy215088-bib-0076] Wheeler, D. S. , Underhill, S. M. , Stolz, D. B. , Murdoch, G. H. , Thiels, E. , Romero, G. , & Amara, S. G. (2015). Amphetamine activates Rho GTPase signaling to mediate dopamine transporter internalization and acute behavioral effects of amphetamine. Proceedings of the National Academy of Sciences, 112, E7138–E7147. 10.1073/pnas.1511670112 PMC4697400 26553986

[phy215088-bib-0077] Wikberg, J. (1977). Release of 3H‐acetylcholine from isolated guinea pig ileum. A radiochemical method for studying the release on the cholinergic neurotransmitter in the intestine. Acta Physiologica Scandinavica, 101, 302–317. 596205 10.1111/j.1748-1716.1977.tb06012.x

[phy215088-bib-0078] Zsilla, G. , Hegyi, D. E. , Baranyi, M. , & Vizi, E. S. (2018). 3,4‐Methylenedioxymethamphetamine, mephedrone, and beta‐phenylethylamine release dopamine from the cytoplasm by means of transporters and keep the concentration high and constant by blocking reuptake. European Journal of Pharmacology, 837, 72–80. 30172789 10.1016/j.ejphar.2018.08.037

